# Impact of different lighting conditions on the tooth-shade selection using intra-oral scanners: An *in-vitro* study

**DOI:** 10.1016/j.heliyon.2024.e38870

**Published:** 2024-10-02

**Authors:** Dusit Nantanapiboon, Porawit Kamnoedboon, Murali Srinivasan

**Affiliations:** a Clinic of General-, Special Care-, and Geriatric Dentistry, Center for Dental Medicine, University of Zurich, Zurich, Switzerland; b Department of Operative Dentistry, Faculty of Dentistry, Chulalongkorn University, Bangkok, 10330, Thailand; c Dental Material Research and Development Center, Faculty of Dentistry, Chulalongkorn University, Bangkok, 10330, Thailand; d Faculty of Dentistry, Chulalongkorn University, Bangkok, 10330, Thailand

**Keywords:** Spectrophotometer, Intra-oral scanner (IOS), Tooth shade selection, Geriatric dentistry

## Abstract

**Objectives:**

To evaluate the accuracy in tooth shade selection under various lighting conditions using different devices, including a color spectrophotometer (CSP) and two intra-oral scanners (IOS: IOS-1 and IOS-2).

**Methods:**

Tooth shade measurements were performed on a standardized shade guide (16 shades, A1-D4) using CSP, IOS-1 and IOS-2. These evaluations were carried out under three lighting conditions: (1) DL - device light source only, (2) RL - DL plus room light, and (3) CL - RL plus dental-chair light. Each shade was measured ten times per lighting condition with each device by a single investigator. Shade detection accuracy was defined as the percentage of matches to the known shade. Logistic regression models were used for statistical analyses (*α* = 0.05).

**Results:**

A total of 1440 measurements were conducted. Lighting conditions of increasing intensity significantly decreased the shade detection accuracy of the devices (CSP: RLvsDL, p > 0.9999, Odds Ratio (OR) = 1; CLvsDL, p = 0.0726, OR = 0.6053, IOS-1: RLvsDL, p < 0.0001, OR = 0.2862; CLvsDL, p < 0.0001, OR = 0.05239 and IOS-2: RLvsDL, p = 0.0002, OR = 0.376; CLvsDL, p < 0.0001, OR = 0.1908). CSP, IOS-1, and IOS-2 had shade detection accuracies of 43.75 %, 74.38 %, and 59.38 % respectively under DL. In RL, IOS-1 and IOS-2 dropped to 50.63 % and 40.63 %, respectively; CSP remained unchanged. Under CL, the accuracy dropped for all devices (CSP: 35.63 %, IOS-1: 20.63 %, IOS-2: 28.75 %). Additionally, the devices demonstrated higher accuracy when measuring shade A compared to shades B, C, and D.

**Conclusions:**

The findings of this in vitro study concluded that the accuracy of tooth shade detection was more reliable, particularly for individuals with shade A, when spectrophotometers and intraoral scanners were used with only their built-in light source. Further purpose-built clinical studies are required to confirm these findings in a clinical context.

## Highlights

• IOSs and CSPs are valuable tools in assisting novice dentists for tooth shade selection.
• For optimal shade accuracy, use these devices with only their built-in light sources and minimize external ambient light.
• When relying solely on their built-in light sources, IOSs are generally more reliable for shade selection than CSPs.
• This advantage of IOSs diminishes with increased exposure to external ambient lighting.
• Shade selection using these devices tends to be more reliable for individuals with tooth shade A.

## 1 Introduction

The principle of tooth restoration involves recreating the form, function, and aesthetics. However, mimicking the natural tooth shade presents challenges due to its unique and complex characteristics. Consequently, accurately assessing tooth shade and appearance can be a meticulous and challenging step for practitioners [1,2].

Tooth shade detection is essential for dental restorative procedures. Various methods exist to achieve the closest match to the natural tooth shade, including visual examination, colorimetry, spectrophotometry, photography, and intraoral scanning. The use of a shade guide in visual examination is a long-established, simple method. However, its accuracy is influenced by numerous factors. These factors include the dentist's experience [3,4], eye fatigue [5], background conditions [2], and ambient lighting [6]. These factors can contribute to discrepancies in clinical outcomes.

Nowadays, various devices have been developed to enhance ease and accuracy in shade selection. These devices consist of an internal light source, such as an LED or laser, which emits light that strikes the object before being absorbed and reflected back to the imaging sensor. The device then analyzes the reflected light and outputs the results in various color systems, such as Vita Classic, Vita 3D Master, and the CIELab L∗, a∗, b∗ value. In these systems, L∗ represents lightness, a∗ corresponds to the red/green coordinate, and b∗ to the yellow/blue coordinate, all of which are beyond the interpretative capacity of the human eye [7]. As demonstrated in a previous study comparing shade selection using human eyes versus spectrophotometers, the results revealed that spectrophotometers exhibited higher accuracy, achieving over 96 % reliability and 67–93 % validity [8].

The development of intraoral scanners was aimed at recording detailed information about teeth and surrounding tissues, while simultaneously capturing tooth shade information. Most studies on intraoral scanners have focused on their accuracy in replicating teeth and soft tissues, comparing them with conventional impression methods and other intraoral scanning devices [[9], [10], [11], [12]]. The result of previous studies had indicated that several factors influence the accuracy of intraoral scanners. These include the scan pattern [13], scanning distance [14], dentist's experience [15], and the tooth preparation characteristics [16]. However, research on the accuracy of tooth shade assessment using intraoral scanners remains limited and has yielded varying results. Some studies suggest that tooth shade assessment with digital intraoral scanners might not be ideal as the primary method in clinical practice, owing to significant differences in color parameters when compared to colorimeters [5]. On the other hand, other studies have demonstrated good accuracy with intraoral scanners, positioning them as high-performance instruments in tooth shade assessment [17,18].

Light plays a crucial role in shade detection. In bright lighting conditions, teeth may appear excessively bright or overly white. Additionally, prolonged exposure to such conditions can lead to eye fatigue for the dentists [6,19]. The impact of lighting on the validity and accuracy of intraoral scanners in shade detection remains inconclusive. A previous study revealed that ambient light influenced the accuracy of both intraoral scanners and spectrophotometers in shade detection, leading to reduced precision [20]. However, subsequent research has shown varying results regarding this matter [3].

Additionally, new intraoral scanners equipped with advanced shade-assessing systems have recently entered the market. The aim of this study is to compare the ability of tooth shade selection under various lighting conditions using two intraoral scanners and a spectrophotometer. The primary null hypothesis posits that different lighting conditions do not affect tooth shade selection by the spectrophotometer and intraoral scanners. The secondary null hypothesis asserts that there are no significant differences in tooth shade selection among these devices.

## 2 Materials and methods

### 2.1 Power analysis

The power analysis size was calculated using G∗Power software version 3.1. The parameters were set with a 95 % confidence interval, 95 % power, and 0.97 effect size. The reference values were taken from a previous study by Revilla-Leon et al. [20]. The total sample size was calculated as at least seven specimens per group. Therefore, this study was designed to use 10 measurements per group.

### 2.2 Tooth shade samples and lighting condition setup

**Fig. 1:**
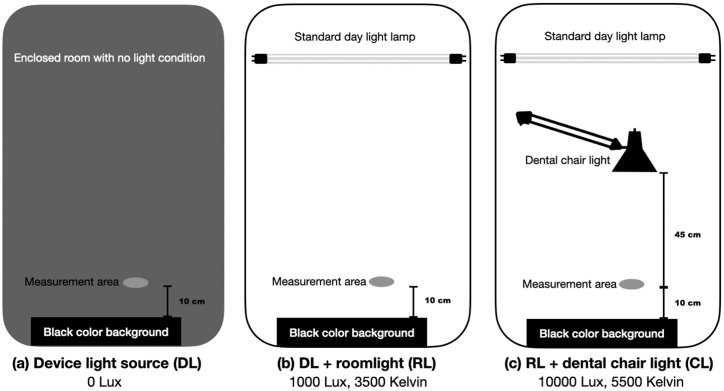
**Illustrative Diagrams of Measurement Settings under Different Lighting Conditions.** This figure presents diagrams depicting the experimental setup for tooth shade selection under various lighting conditions with controlled light intensity and color temperature. **(a)** Device Light Source setting, labelled as “DL”, showing the measurement in a dark environment with only the device's light source active; **(b)** Device Light Source plus Room Light setting, labelled as “RL”, demonstrating the arrangement in a typical dental treatment room with standard room lighting; and **(c)** Device Light Source plus Room Light and Dental-Chair Light setting, labelled as “CL”, depicting the measurement conducted with additional illumination from a dental-chair light source.

In this study, 16 shades tabs (A1-D4) from a shade guide (VITA classical, VITA Zahnfabrik, Bad Säckingen, Germany) were utilized as standardized tooth-shade samples. The experiment was conducted under three distinct lighting scenarios: device light source (DL), device light source plus room light (RL), and device light source plus room light plus dental-chair light (CL), as illustrated in Fig. 1.

Under the DL condition, tooth shade selection took place in a darkened room devoid of windows and with all lights off, relying solely on the built-in device light source for illumination. In the RL setting, the procedure was carried out in a standard dental treatment room (dimensions: 4x4x3 meters) equipped with 18 standard daylight lamps (TL-D 90 Graphica 36W/965 Philips, Amsterdam, Netherlands). The controlled working area within the room was maintained with a light intensity of 1000 lux and a color temperature of 3500 K.

For the CL condition, tooth shade selection occurred under the same room conditions as RL, but with additional illumination from a dental unit light source (LEDView Plus, Dental Chair Sinius, Sirona Dental Systems GmbH, Bensheim, Germany). The setup in this scenario ensured a light intensity of 10000 lux and a color temperature of 5500 K in the controlled working area. The distance between the dental unit light source and the shade tabs was consistently maintained at 45 cm to mimic the typical distance between a patient's tooth and the dental unit light in clinical settings.

### 2.3 Devices and operation

This study employed three devices with tooth shade selection functions: a color spectrophotometer (CSP: Vita Easyshade®V, VITA Zahnfabrik, Bad Säckingen, Germany) and two intraoral scanners (IOS-1: Trios4 Intraoral Scanner, 3Shape A/S, Copenhagen, Denmark; and IOS-2: Cerec Primescan, Sirona Dental Systems, Bensheim, Germany). All tooth shade selections were performed by a trained operator (DN). For each shade tab, ten separate measurements were taken under various lighting conditions using each device. Before measuring each new shade tab, the devices were calibrated in accordance with the manufacturer's guidelines.

#### 2.3.1 Color spectrophotometer (CSP)

CSP was used for shade detection, adhering to the manufacturer's instructions in the basic shade measurement mode. Calibration was conducted prior to each new measurement session for each shade tab. During the measurement, the scanner's tip was positioned at a 90° angle near the center of the shade tab, specifically in the middle third area. This process was replicated ten times for each shade tab across all lighting conditions, and the measured shades were documented as VITA classic shades for subsequent analysis.

#### 2.3.2 Intraoral scanners (IOS-1 and IOS-2)

**Fig. 2:**
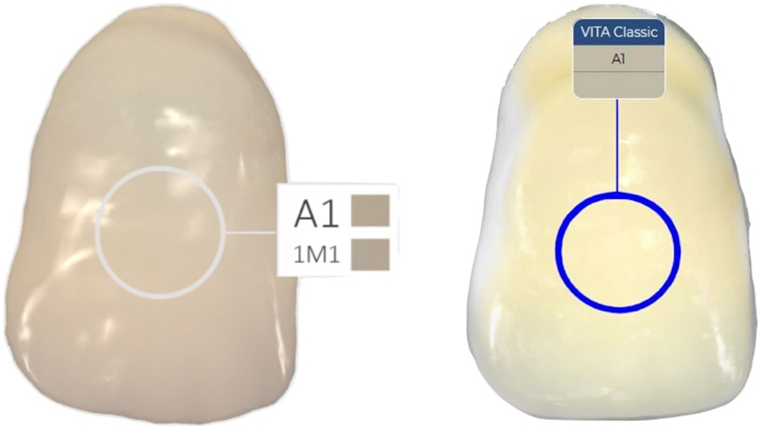
**Shade Determination Area.** This figure highlights the middle third area of the tooth shade tab, indicating the region after scanned by the intraoral scanners. This consistent focal area was used for each device across all shade tabs and under various lighting conditions.

For IOS-1, color calibration was followed by a scanning process that started at the top of the shade tab, moving the scanner head in a mesio-distal motion for 20 s. The scanned data was then analyzed using 3Shape Unite software version 23.1 (3Shape A/S, Copenhagen, Denmark) to determine the tooth shade in the VITA classic shade, choosing on the middle third area of the shade tab, as shown in Fig. 2. This procedure was repeated ten times for each shade tab under all lighting conditions. The same protocol was followed for IOS-2, with the analysis conducted using Cerec software version 5.5.1.

### 2.4 Data and statistical analysis

The tooth shade data obtained from each device were compared to the corresponding measured tooth shade tab. A match between the device's recorded shade and the measured shade tab was classified as “Correct.” Conversely, any discrepancy between the two was categorized as “Incorrect.” The accuracy of each device's shade determination was quantified as the percentage of correct matches to the known shade, representing the proportion of “Correct” outcomes in the total measurements.

Prism10 software for macOS, version 10.1.1 (270), was utilized. The analysis employed multiple logistic regression models, an appropriate choice given the binary nature of the outcomes (correct/incorrect) and the multiple categorical predictors (devices, shades). This method allows for the assessment of the influence of lighting conditions on tooth shade selection accuracy while controlling for the effects of multiple predictors [21,22]. The choice of this model is further justified as it accounts for potential confounding factors and provides a robust analysis of the comparative performance of the devices [23]. A significance level (**α**) was set at 0.05, providing a threshold for determining statistical significance in the study's findings.

To assess the consistency and reliability among the repeated measurements for each shade tab across different lighting conditions and devices, Fleiss' Kappa was calculated using IBM SPSS software, version 28.0.1.1 (14). Fleiss' Kappa is a statistical measure of inter-rater reliability that accounts for the agreement between multiple measurements, beyond what would be expected by chance [24,25]. This analysis was conducted across three lighting conditions (DL, RL, and CL), for the three devices (CSP, IOS-1, and IOS-2). Fleiss' Kappa values, along with their 95 % confidence intervals, were computed to quantify the level of agreement in shade determination across repeated measurements.

## 3 Results

### 3.1 Descriptive statistical analysis of device accuracy across various lighting conditions

The study evaluated outcomes across various lighting conditions (device light source (DL), DL + room light (RL), and RL + dental-chair light (CL)) and tooth-color shades (A1-D4), with a total of 1440 measurements. Table 1 summarizes the accuracy of each device in determining tooth shade under these conditions and shades. Complete raw data is available in Appendix A.

**Table 1:**
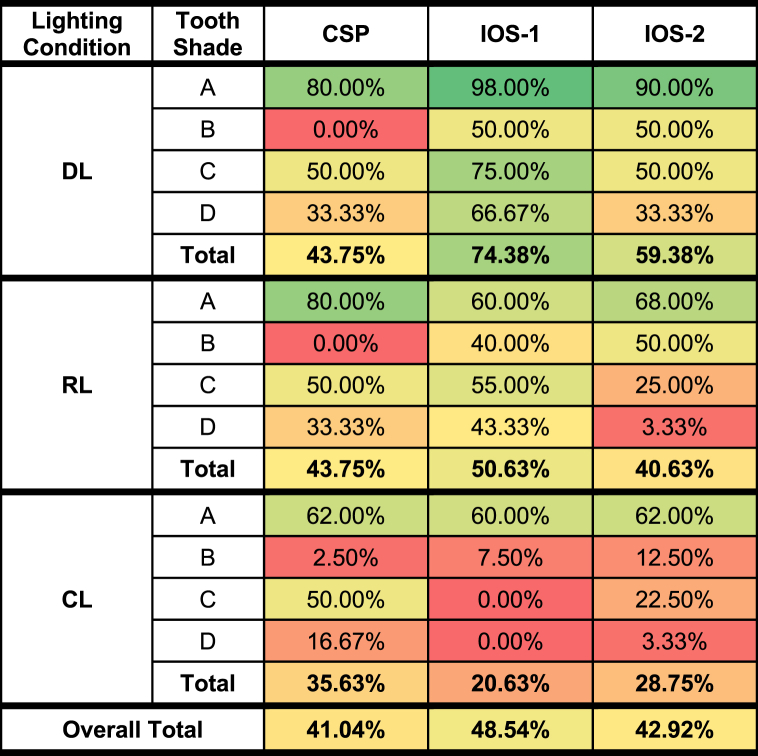
Distribution of Tooth Shade Selection Accuracies of each Device by Lighting Conditions and Tooth-Color Shades.

The table outlines the accuracy of tooth shade determinations for each device, represented as the percentage of correct matches to known shades. The measurements were taken using a color spectrophotometer (CSP, VitaEasyshade) and two intra-oral scanners (IOS-1, Trios4; IOS-2, PrimeScan). Results are presented across various lighting conditions—device light source only (DL), device light plus room light (RL), and room light combined with dental-chair light (CL)—and for different tooth-color shades (Shades A, B, C, and D).

Under DL conditions, the color spectrometer (CSP) achieved an overall accuracy of 43.75 %, with 80 % accuracy for A shades, but lower accuracy for B, C, and D shades (0 %, 50 %, and 33.33 %, respectively). The Trios4 scanner (IOS-1) had a 74.38 % overall accuracy, with 98 % for A shades and 50 %, 75 %, and 66.67 % for B, C, and D shades. The Cerec PrimeScan (IOS-2) showed 59.38 % overall accuracy, with 90 % for A shades and 50 %, 50 %, and 33.33 % for B, C, and D shades. In the RL setting, CSP's overall accuracy remained at 43.75 %, while IOS-1 and IOS-2's accuracies dropped to 50.63 % and 40.63 %, respectively, with A shades showing the highest accuracy for both devices. Under CL conditions, CSP's accuracy declined to 35.63 %, IOS-1's to 20.63 %, and IOS-2's to 28.75 %. A shades provided the most reliable detection across all devices.

### 3.2 Analysis the impact of lighting conditions on each device in tooth shade selection

Multiple logistic regression models, as shown in Table 2, reveal the impact of lighting conditions and tooth shades on device performance. For CSP, the intercept showed a significant odds ratio (OR = 3.409, P < 0.0001). RL had little effect (OR = 1, P > 0.9999), while CL reduced the odds slightly (OR = 0.6053, P = 0.0726). Shades B, C, and D significantly lowered the odds compared to Shade A.

**Table 2:** Multiple logistic regression analysis for the impact of lighting conditions and tooth shades on each device.

	Variable	Parameter estimate (SE)	Odds ratio (95 % CI)	P value
CSP				
	Intercept	1.226 (0.2517)	3.409 (2.107–5.665)	**<0.0001**
	Lighting[RL]	4.265E-17 (0.2801)	1 (0.5770–1.733)	>0.9999
	Lighting[CL]	−0.502 (0.2796)	0.6053 (0.3484–1.044)	0.0726
	Shade[B]	−5.864 (1.022)	0.002839 (0.0001586–0.01344)	**<0.0001**
	Shade[C]	−1.06 (0.2628)	0.3465 (0.2057–0.5771)	**<0.0001**
	Shade[D]	−2.027 (0.3027)	0.1317 (0.07163–0.2353)	**<0.0001**
**IOS-1**				
	Intercept	2.733 (0.309)	15.38 (8.579–28.85)	**<0.0001**
	Lighting[RL]	−1.251 (0.2628)	0.2862 (0.1695–0.4757)	**<0.0001**
	Lighting[CL]	−2.949 (0.3138)	0.05239 (0.02769–0.09501)	**<0.0001**
	Shade[B]	−2.324 (0.3267)	0.09788 (0.05059–0.1826)	**<0.0001**
	Shade[C]	−1.721 (0.3137)	0.1789 (0.09518–0.3266)	**<0.0001**
	Shade[D]	−2.087 (0.3442)	0.124 (0.06202–0.2398)	**<0.0001**
**IOS-2**				
	Intercept	1.998 (0.2695)	7.373 (4.416–12.72)	**<0.0001**
	Lighting[RL]	−0.978 (0.2614)	0.376 (0.2237–0.6243)	**0.0002**
	Lighting[CL]	−1.656 (0.2777)	0.1908 (0.1094–0.3255)	**<0.0001**
	Shade[B]	−1.683 (0.2813)	0.1857 (0.1058–0.3193)	**<0.0001**
	Shade[C]	−1.927 (0.2872)	0.1455 (0.08181–0.2527)	**<0.0001**
	Shade[D]	−3.159 (0.3825)	0.04245 (0.01926–0.08691)	**<0.0001**

The table presents the parameter estimates from multiple logistic regression models predicting the outcome for each device based on lighting conditions and tooth-color shades. The reference levels were set to the “DL” for lighting condition variable and the “Shade A” for tooth-color shade variable. CSP, VitaEasyshade; IOS-1, Trios4; IOS-2, PrimeScan; DL, device light source only; RL, DL + room light; CL, RL + dental-chair light; SE, standard error; CI, confidence interval.

IOS-1 had a higher baseline probability (OR = 15.38, P < 0.0001), but both RL and CL significantly decreased the odds (OR = 0.2862 and OR = 0.05239, respectively, P < 0.0001). Shades B, C, and D also reduced the odds substantially compared to Shade A.

For IOS-2, the intercept indicated a moderate probability (OR = 7.373, P < 0.0001). RL and CL significantly reduced the odds (OR = 0.376 and OR = 0.1908, respectively, P < 0.0001). Shades B, C, and D further decreased the likelihood of correct shade determination, with Shade D having the strongest negative effect.

Overall, the analysis showed that additional lighting, especially with the dental-chair light, generally decreased the accuracy of shade determination. Across all devices, deviations from Shade A resulted in progressively lower accuracy, with Shade A yielding the most reliable outcomes.

### 3.3 Comparative analysis of device performance across lighting conditions

The findings from Table 3, ascertained through multiple logistic regression analyses, provided further insights into the comparative performance among different devices under specific lighting conditions. Under DL, the intercept was not significant (OR = 0.7778, P = 0.1148), but IOS-1 significantly outperformed CSP (OR = 3.857, P < 0.0001), with IOS-2 also showing a smaller increase in odds (OR = 1.879, P = 0.0054).

**Table 3:** Multiple logistic regression analysis for device performance across lighting conditions.

	Variable	Parameter estimate (SE)	Odds ratio (95 % CI)	P value
DL				
	Intercept	−0.2513 (0.1594)	0.7778 (0.5676–1.061)	0.1148
	Device[IOS-1]	1.35 (0.2423)	3.857 (2.414–6.250)	**<0.0001**
	Device[IOS-2]	0.6308 (0.2265)	1.879 (1.208–2.938)	**0.0054**
**RL**				
	Intercept	−0.2513 (0.1594)	0.7778 (0.5676–1.061)	0.1148
	Device[IOS-1]	0.2763 (0.2245)	1.318 (0.8496–2.050)	0.2184
	Device[IOS-2]	−0.1282 (0.2265)	0.8797 (0.5637–1.371)	0.5715
**CL**				
	Intercept	−0.5917 (0.1651)	0.5534 (0.3981–0.7615)	**0.0003**
	Device[IOS-1]	−0.756 (0.2558)	0.4695 (0.2823–0.7712)	**0.0031**
	Device[IOS-2]	−0.3159 (0.2403)	0.7291 (0.4540–1.166)	0.1887

The table presents the parameter estimates from multiple logistic regression models predicting the outcome for each lighting condition. The reference level was set to the “CSP, VitaEasyshade”. IOS-1, Trios4; IOS-2, PrimeScan; DL, device light source; RL, DL + room light; CL, RL + dental-chair light; SE, standard error; CI, confidence interval.

In RL, the baseline remained similar to DL, with IOS-1 showing a modest, non-significant increase (OR = 1.318, P = 0.2184), and IOS-2 showing a slight, non-significant decrease (OR = 0.8797, P = 0.5715).

Under CL, the intercept indicated a significantly lower likelihood of success (OR = 0.5534, P = 0.0003). IOS-1 performed worse than CSP (OR = 0.4695, P = 0.0031), and IOS-2 also showed a reduction, though not significant (OR = 0.7291, P = 0.1887).

This logistic regression analysis highlighted that, in specific lighting condition, which device performed more reliably in tooth shade selection. In the DL condition, both IOS-1 and IOS-2 showed increased odds of the outcome compared to CSP. However, under RL, the impact of device choice was less pronounced. In contrast, the CL condition showed a generally lower likelihood of achieving the expected outcome, with both IOS-1 and IOS-2 less favorable than CSP.

### 3.4 Reliability tests

As depicted in Table 4, CSP consistently showed almost perfect to substantial reliability across all lighting conditions, particularly excelling under DL and RL. IOS-1 exhibited strong reliability under DL and CL, but its performance diminished significantly under RL. IOS-2 demonstrated substantial reliability under DL and RL, but its reliability dropped to moderate levels under CL.

**Table 4:** Fleiss' Kappa values for reliability of tooth shade selection across different devices and lighting conditions.

	CSP	IOS-1	IOS-2
DL	1.000 [0.927–1.073]	1.000 [0.927–1.073]	0.882 [0.809–0.955]
RL	1.000 [0.927–1.073]	0.525 [0.452–0.598]	0.680 [0.607–0.753]
CL	0.870 [0.797–0.943]	0.818 [0.745–0.891]	0.417 [0.344–0.490]

Fleiss' Kappa values with 95 % confidence intervals are shown for each device under three lighting conditions. Fleiss' Kappa values closer to 1 indicate higher reliability of the device in consistently measuring tooth shades across repeated measurements. IOS-1, Trios4; IOS-2, PrimeScan; DL, device light source; RL, DL + room light; CL, RL + dental-chair light.

## 4 Discussion

In this study, we conducted a comprehensive comparison of tooth shade selection under various lighting conditions using two intraoral scanners and a color spectrophotometer. Our findings indicated that lighting conditions significantly influence tooth shade selection, leading to the rejection of our primary null hypothesis. Specifically, we observed that the accuracy of devices varied depending on the lighting setup, with a notable decrease in accuracy under room light and dental-chair light conditions compared to device light only condition. Additionally, the secondary null hypothesis was also rejected, as there were discernible differences in tooth shade selection among the different devices. In the device light only condition, both intraoral scanners performed better in tooth shade matching compared to the color spectrophotometer. However, under room light condition, the difference in performance between the devices was less noticeable. In contrast, in the dental-chair light setting, the likelihood of accurately determining tooth shade was generally lower, with both intraoral scanners performing less effectively than color spectrophotometer.

The determination of tooth shade is a crucial step that can significantly impact a patient's aesthetic treatment outcome. While visual examination is a straightforward method, there is inherent uncertainty in the results due to several factors that can influence tooth shade detection. These factors include the dentist's experience [3], eye fatigue [5], variations in shade tabs [26], background conditions [2], and ambient light [6]. Previous research had indicated that using a spectrophotometer yields more accurate and repeatable results compared to visual evaluation [27,28]. Therefore, our study did not utilize visual evaluation as a comparative standard. We defined device accuracy as validity in shade detection, referring to the ability to accurately detect tooth shade [29]. Ideally, the tooth shade values derived from the devices should align with the shade tab, ensuring consistent and repeatable results.

In dental shade detection research, many researchers commonly employed natural teeth, porcelain discs, or shade tabs as sample representations of various shades. However, the natural teeth were complicated subjects influenced by multiple factors, including moisture, age of the teeth, and measurement location, all of which can significantly impact the result of tooth shade detection. This study simulated diverse clinic lighting conditions, recognizing that intense light can induce tooth dehydration, consequently impacting the observed tooth shade [30]. Although some studies used porcelain discs as samples in their studies, a previous study showed no significant difference when using 1-mm porcelain discs or shade tabs as references, indicating that they are interchangeable for assessing detection reliability and accuracy [31]. To ensure the standards in tooth shade detection, this study utilized the Vita Classic shade guide as a standardized tooth shade reference, maintaining uniformity across 16 shade tabs. This methodology conforms to ISO/TR 28642:2016, affirming the accuracy and reliability in tooth shade measurement [32].

Vita Easyshade®V serves as a widely utilized spectrophotometer for tooth shade detection in both research and clinical settings. It's esteemed for its easy-to-use, high accuracy, repeatability and less influenced by surrounding ambient light. This device had been recommended as a gold standard in tooth shade detection due to its higher precision compared to other devices and its superior to human eye, as indicated in various studies [29,33,34]. The device utilized its internal light source, interacting with the object under tooth shade detection and capturing the reflected light. Despite its advantages, Vita Easyshade®V has limitations, particularly in the size of its large diameter measuring head and the inherent curvature of dental anatomy. These factors can challenge related to light reflection, particularly in scenarios involving small-sized teeth or dental prostheses. All these considerations make the device technique sensitive and contribute to potential inaccuracies in measurements.

An intraoral scanner was first developed for recording the teeth characteristics and surrounding tissues instead of using the conventional impression. The intraoral scanner could reduce the treatment procedures and time with better satisfaction from the patients. In this study, the intraoral scanner Trios4 and Cerec PrimeScan were included. The Trios4 utilized confocal laser technology for data capturing that relies on the acquisition of focused and defocused images from selected areas. This technology detected the sharpness area of the image to infer the distance to the object, correlated to the focal length of the lens [35]. On the other hand, Cerec PrimeScan relies on triangulation of video capturing technology to record the data and can generate a 3D-colored image [36,37]. The shade selection of intraoral scanner could also be conducted by using the same principle as an image editing program. The point needed for shade selection is chosen and the program will then analyze and evaluate to report the most similar color as the result. Previous studies revealed that the efficacy in tooth shade matching by using the intra-oral scanners was comparable to a spectrophotometer (Vita Easyshade®V) and demonstrated significantly superior results compared to human visual assessment [33]. Furthermore, in a study conducted by Ebeid et al., results revealed no statistically significant difference in accuracy for tooth shade detection between Vita Easyshade®V (78 %) and TRIOS 3 intra-oral scanner (66 %). Nonetheless, significant differences were observed with Cerec PrimeScan (63 %) and Cerec Omnicam (57 %) and both of all showed low overall accuracy (<80 %) [38].

Light significantly influences a dentist's work environment, with recommendations emphasizing visibility without excessive brightness. Light temperature, measured in Kelvin (K), determines its color tone: lower light temperatures result in warmer, yellowish light, while higher light temperature creates cooler, bluish tones. This color temperature scale is crucial as it affects how colors appear under various lighting conditions, influencing perception and color accuracy in assessment. In alignment with the European Committee for Standardization of light and lighting of workplaces [39], this study incorporated light intensity and temperature as variables, representing three distinct clinical conditions. The first condition, the device light source condition, simulated using only the device's light source or a room with no external light source. The room light condition, represented by 1000 Lux and 3500 K, reflected the lighting conditions of dental examination rooms. Additionally, the dental operation room is ensured to be well-lit with a recommended intensity ranging from 10000 to 20000 lux, as illustrated in the dental chair light condition at 10000 Lux and 5500 K.

From the result of this study, the most precise tooth shade detection was achieved under device light only condition, wherein illumination solely originates from the tooth shade detection devices. The exposure of external ambient light sources, as observed in the room light or dental-chair light conditions, notably decreased the accuracy of intra-oral scanners. However, the Vita Easyshade®V only showed decreased under the dental-chair light condition. These findings correlated to Revilla-Leon et al.’s research [20], the significant impact of ambient light sources on shade matching between spectrophotometer and intraoral scanners. They emphasized that the absence of light (0 Lux) showed the highest consistency in the Vita Easyshade®V and the Trios3 intra-oral scanner. As light intensity increased, the accuracy of tooth shade detection decreases notably in the Trios3 intra-oral scanner. Particularly, this effect was noticeable in the Vita Easyshade®V only at the 10000-lux light intensity. Contrarily, Yilmaz et al.’ study [3] reported no impact on accuracy when utilizing Vita Easyshade®V and Trios3 intra-oral scanner with light temperatures of 4000 K and 6500 K. This discrepancy might be attributed to factors such as a small sample size and the limited range of tooth shades among patients.

Based on the results of this study, we recommend a comprehensive approach using scanner-assisted methods in combination with other methods, such as visual examination, for tooth shade selection. Furthermore, all tested devices demonstrated a higher percentage of shade matching in the A shade compared to other shades, indicating a more reliable shade determination for patients with shade A teeth.

The limitation of this study was that it could not represent clinical situations like tooth shade detection in patients. The research used shade tabs instead of natural teeth to reduce factors related to teeth moisture and to achieve a wide range of teeth colors. Moreover, the study did not include daylight as a lighting condition due to its variability, which can be influenced by factors such as time of day, weather conditions, and the orientation of the room relative to the sun, all of which affect color temperature and light intensity. Room lighting, by contrast, offers a more controlled and repeatable environment for experimental purposes. Further research could employ a clinical study and should include more patients with a broader range of tooth shades. Additionally, further studies might evaluate tooth shade comparison using other devices such as photography with shade-matching software or new intraoral scanner with updated versions of shade-matching functions.

## 5 Conclusions

Considering the limitations of this study, the following conclusions can be drawn.

• Light significantly impacts on tooth shade selection of the devices.
• The accuracy of tooth shade detection, by spectrophotometers and intra-oral scanners, is more reliable when using only the built-in light source of the respective devices.
• When relying solely on their built-in light sources, intraoral scanners are generally more reliable for tooth shade selection than spectrophotometers. However, this advantage diminishes with increased exposure to external ambient lighting.
• Tooth shade selection using the devices tends to be more reliable for individuals with shade A compared to those with shades B, C, and D.

## Funding

This research did not receive any specific grant from funding agencies in the public, commercial, or not – for – profit sectors.

## Data availability statement

The datasets used and analyzed during the current study are available from the corresponding author on request.

## CRediT authorship contribution statement

**Dusit Nantanapiboon:** Writing – review & editing, Writing – original draft, Visualization, Methodology, Investigation, Data curation, Conceptualization. **Porawit Kamnoedboon:** Writing – review & editing, Writing – original draft, Methodology, Investigation, Formal analysis, Data curation, Conceptualization. **Murali Srinivasan:** Writing – review & editing, Supervision, Resources, Methodology, Conceptualization.

## Declaration of competing interest

The authors declare that they have no known competing financial interests or personal relationships that could have appeared to influence the work reported in this paper.
