# Supplementary material for: Impact of different lighting conditions on the tooth-shade selection using intra-oral scanners: An in-vitro study
Source: Heliyon. 2024 Oct 2;10(19):e38870. doi: 10.1016/j.heliyon.2024.e38870 (PMC11491899; doi:10.1016/j.heliyon.2024.e38870)
Supplement: Multimedia component 1 [file mmc1.docx]

**Appendix A**. Distribution of Tooth Shade Determination Accuracies of each Device by Lighting Conditions and Tooth-Color Shades

| **Lighting Condition** | **Tooth Shade** | **CSP** | | **IOS-1** | | **IOS-2** | |
| --- | --- | --- | --- | --- | --- | --- | --- |
|  |  | **Correct** | **Incorrect** | **Correct** | **Incorrect** | **Correct** | **Incorrect** |
| **DL** | A | 40  (80.00%) | 10  (20.00%) | 49  (98.00%) | 1  (2.00%) | 45  (90.00%) | 5  (10.00%) |
|  | B | 0  (0.00%) | 40  (100.00%) | 20  (50.00%) | 20  (50.00%) | 20  (50.00%) | 20  (50.00%) |
|  | C | 20  (50.00%) | 20  (50.00%) | 30  (75.00%) | 10  (25.00%) | 20  (50.00%) | 20  (50.00%) |
|  | D | 10  (33.33%) | 20  (66.67%) | 20  (66.67%) | 10  (33.33%) | 10  (33.33%) | 20  (66.67%) |
|  | **Total** | **70**  **(43.75%)** | **90**  **(56.25%)** | **119**  **(74.38%)** | **41**  **(25.63%)** | **95**  **(59.38%)** | **65**  **(40.63%)** |
| **RL** | A | 40  (80.00%) | 10  (20.00%) | 30  (60.00%) | 20  (40.00%) | 34  (68.00%) | 16  (32.00%) |
|  | B | 0  (0.00%) | 40  (100.00%) | 16  (40.00%) | 24  (60.00%) | 20  (50.00%) | 20  (50.00%) |
|  | C | 20  (50.00%) | 20  (50.00%) | 22  (55.00%) | 18  (45.00%) | 10  (25.00%) | 30  (75.00%) |
|  | D | 10  (33.33%) | 20  (66.67%) | 13  (43.33%) | 17  (56.67%) | 1  (3.33%) | 29  (96.67%) |
|  | **Total** | **70**  **(43.75%)** | **90**  **(56.25%)** | **81**  **(50.63%)** | **79**  **(49.38%)** | **65**  **(40.63%)** | **95**  **(59.38%)** |
| **CL** | A | 31  (62.00%) | 19  (38.00%) | 30  (60.00%) | 20  (40.00%) | 31  (62.00%) | 19  (38.00%) |
|  | B | 1  (2.50%) | 39  (97.50%) | 3  (7.50%) | 37  (92.50%) | 5  (12.50%) | 35  (87.50%) |
|  | C | 20  (50.00%) | 20  (50.00%) | 0  (0.00%) | 40  (100.00%) | 9  (22.50%) | 31  (77.50%) |
|  | D | 5  (16.67%) | 25  (83.33%) | 0  (0.00%) | 30  (100.00%) | 1  (3.33%) | 29  (96.67%) |
|  | **Total** | **57**  **(35.63%)** | **103**  **(64.38%)** | **33**  **(20.63%)** | **127**  **(79.38%)** | **46**  **(28.75%)** | **114**  **(71.25%)** |
| **Overall Total** | | **197**  **(41.04%)** | **283**  **(58.96%)** | **233**  **(48.54%)** | **247**  **(51.46%)** | **206**  **(42.92%)** | **274**  **(57.08%)** |

The table delineates the distribution of outcomes, categorized as "Correct" and "Incorrect," as measured by the color spectrophotometer (CSP, VitaEasyshade) and two intra-oral scanners (IOS-1; Trios4; IOS-2, PrimeScan). These outcomes are presented across different lighting conditions: device light source (DL), DL + room light (RL), and RL + dental-chair light (CL) and tooth-color shades (Shades A, B, C, and D). Each cell provides the raw count and corresponding percentage (%) of the total for that category. The total rows represent the combined influence of lighting conditions and tooth-color shades on the outcomes.
